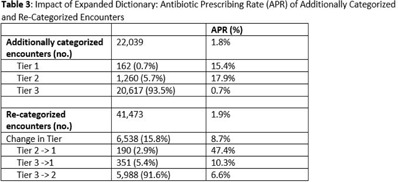# Metrics in outpatient stewardship: Is more always better?

**DOI:** 10.1017/ash.2022.192

**Published:** 2022-05-16

**Authors:** Natalia Medvedeva, David Ha, Sharon Onguti, Emily Rosen, Emily Mui, Sean Pearce, Alex Schneider, Amy Chang, Adam Hersh, Eddie Stenehjem, Marisa Holubar

## Abstract

**Background:** Emerging evidence supports the use of billing data to identify stewardship targets in primary care. Standardizing an approach to antibiotic prescribing rate (APR) calculations could facilitate external benchmarking. **Methods:** Using methodology and an ICD-10 dictionary validated in urgent care clinics,^1^ we created an expanded ICD-10 dictionary to incorporate additional ICD-10 codes from primary care associated with antibiotic prescriptions (Fig. 1). We then compared antibiotic prescribing rates using the urgent care and expanded dictionaries. We included all primary care visits from 2019 to 2020 and extracted ICD-10 codes and antibiotic order data. Using the urgent care and expanded ICD-10 dictionary, we classified each encounter by prescribing tier based on whether antibiotics are almost always (tier 1), sometimes (tier 2), or almost never (tier 3) indicated. For encounters with ICD-10s in multiple tiers, we chose the lowest tier. For multiple ICD-10 codes within the same tier, we chose the first extracted ICD-10 code. We calculated antibiotic prescribing rates as the proportion of encounters associated with ≥ 1 antibacterial prescription. This quality improvement project was deemed non–human subjects research by the Stanford Panel on Human Subjects in Medical Research. **Results:** The urgent care dictionary has 1,400 ICD-10 codes. We added 1,439 ICD-10 codes derived from primary care encounters to create the expanded ICD-10 dictionary (8.5% tier 1, 9.1% tier 2, and 82.4% tier 3) (Fig. 1). We identified 177,531 encounters; 74% had ≥ 2 associated ICD-10 codes (Fig. 2). In total, 147,085 encounters (82.9%) were classified into a tier using the urgent care dictionary. An additional 22,039 encounters were classified with the expanded dictionary (Table 1). Most added encounters were tier 3 with low 0.7% APR (Tables 1 and 3). In total, 41,473 (28.2%) encounters were classified differently depending on the ICD-10 dictionary used, most commonly changing from tier 3 to tier 2 without an increase in overall tier 2 antibiotic prescribing rate (Tables 2 and 3). Overall antibiotic prescribing rates were similar when using either the urgent care or expanded ICD-10 dictionary (Table 2). **Conclusions:** The expanded ICD-10 dictionary allowed for classification of more encounters in primary care; however, it did not meaningfully change antibiotic prescribing rates. Antibiotic prescribing rates were likely diluted by classifying more encounters without identifying an associated increase in antibiotic prescribing. A more sophisticated classification system may help to accommodate the diversity and volume of ICD-10 codes used in primary care.

1. Stenehjem E, et al. *Clin Infect Dis* 2020;70:1781–1787.

**Funding:** None

**Disclosures:** None

**Figure:**
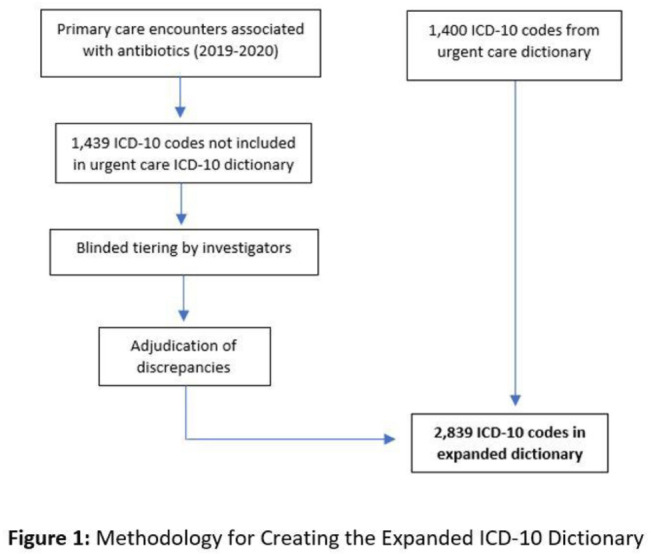

**Figure:**
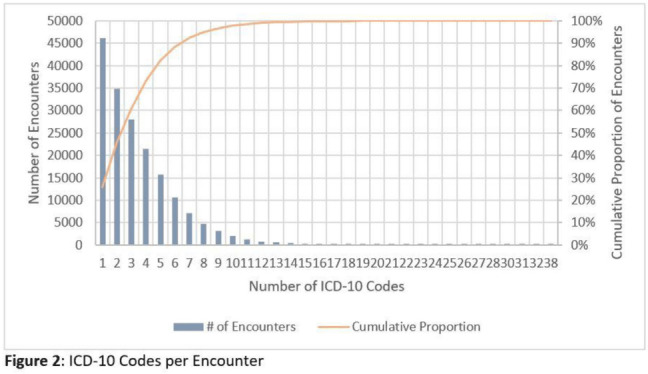

**Table:**
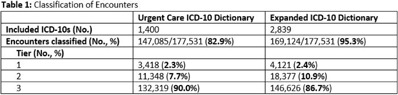

**Table:**
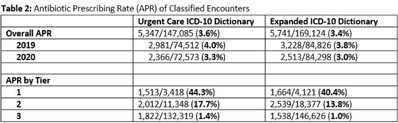

**Table:**